# validation of a scale for assessing patient satisfaction with the quality of care received in psychiatric settings

**DOI:** 10.1192/j.eurpsy.2022.1515

**Published:** 2022-09-01

**Authors:** K. Razki, Y. Zgueb, A. Aissa, U. Ouali, R. Jomli

**Affiliations:** Hôpital Razi, Service Psychiatrie A, La Manouba, Tunisia

**Keywords:** validation, psychiatric care, patient perception, satisfaction scale

## Abstract

**Introduction:**

The complexity of the feeling of satisfaction makes its measurement complex, in this context our work aims to develop a simple and practical measurement tool to identify problems within the processes of psychiatric care in order to provide corrective interventions.

**Objectives:**

to validate the psychometric properties of a scale designed for us to assess patients’ satisfaction with the quality of psychiatric care received.

**Methods:**

This is a validation study conducted on a sample of 200 patients followed at RAZI Hospital in Tunisia, outside any period of hospitalization. The questionnaire consisted of 28 items and was structured around eight dimensions (the patient’s perception of his or her own mental disorder, the quality of the doctor-patient relationship, the quality of the nursing team-patient relationship, the organisational aspect and conditions of the hospital ward, the therapeutic discharge planning, the respect of human rights, and the quality of the patient’s health, The organisational aspect and conditions of the hospital ward, Therapeutic discharge planning, Respect for patients’ human rights, Satisfaction with overall care and Loyalty.

**Results:**

Both face validity and content validity were satisfactory. Internal consistency was sufficient with a Cronbach’s alpha of 0.913. The inter-dimensional correlation reflected statistically significant and logical correlations within our scale. Temporal stability was satisfactory. An exploratory factor analysis revealed seven factors with a Kaiser-Meyer-Olkin score of 0.852.

**Conclusions:**

Our scale has demonstrated good psychometric properties. It can be reliably used as a measure of the satisfaction of Tunisian patients with the psychiatric care received.

**Disclosure:**

No significant relationships.

